# Detection of ALK protein expression in lung squamous cell carcinomas by immunohistochemistry

**DOI:** 10.1186/s13046-014-0109-2

**Published:** 2014-12-21

**Authors:** Jiandong Wang, Qin Shen, Qunli Shi, Bo Yu, Xuan Wang, Kai Cheng, Guangming Lu, Xiaojun Zhou

**Affiliations:** Department of Pathology, Jinling Hospital, Nanjing University School of Medicine, Nanjing, 210002 China; Department of Radiology, Jinling Hospital, Nanjing University School of Medicine, Nanjing, 210002 China

**Keywords:** Anaplastic lymphoma kinase, Lung squamous cell carcinoma, IHC, D5F3 clone

## Abstract

**Background:**

The echinoderm microtubule-associated protein-like 4 (EML4) gene and the anaplastic lymphoma kinase (ALK) gene rearrangements occur in approximately 5% of lung adenocarcimomas (ACA), leading to ALK overexpression and predicting response to targeted therapy. To the present, few studies have been focused on the expression of ALK protein in lung squamous cell carcinomas (SqCC). Only several cases of lung SqCC were reported expression of ALK protein. No clinical study has been published to explicit the relationship between ALK expression and the response to targeted therapy in SqCC.

**Methods:**

In this study, we analyzed ALK protein expression with a specific rabbit monoclonal Ig antibody (D5F3 clone) in 207 cases of lung SqCC. The positive cases were confirmed with ALK fluorescence in situ hybridization (FISH) and RT-PCR.

**Results:**

We found that 3 out of 207 (1.4%) cases of lung SqCC were ALK positive detected by IHC staining, which were confirmed by ALK FISH and RT-PCR.

**Conclusions:**

Our results indicate that ALK protein expression is not a rare molecular event in SqCC. Although the frequency of EML4-ALK rearrangements is lower in lung SqCC than that in lung adenocarcinomas, their presence may provide additional treatment options in lung SqCC. The response of SqCC patients with ALK expression to target therapy of crizotinib should be explored.

**Electronic supplementary material:**

The online version of this article (doi:10.1186/s13046-014-0109-2) contains supplementary material, which is available to authorized users.

## Background

Despite the extensive research and clinical efforts dedicated to the management of lung cancer in the past decade, lung cancer remains the leading cause of cancer-related mortality in China and Western countries [1-4]. Patients with lung cancer account for approximately 1.4 million deaths per year worldwide and approximately 160,000 deaths per year in the United States [5]. Histologically, lung cancer is dichotomized into small cell lung carcinoma and non-small cell lung carcinoma (NSCLC). NSCLCs represent a diverse entity that can be subclassified further into distinct histologic subtypes including adenocarcinoma, squamous cell carcinoma (SqCC), large cell carcinoma, large cell neuroendocrine carcinoma, anaplastic carcinoma, and giant cell carcinoma. NSCLCs represent approximately 80% of all lung cancer subtypes and it is the leading worldwide cause of cancer related death. Lung SqCC is a common type of NSCLCs, causing approximately 400,000 deaths per year worldwide. Most lung cancers are often diagnosed at advanced stages of the disease, and the mainstay of therapy is systemic chemotherapy, typically with a platinum-based regimen. Given that conventional chemotherapeutic regimens only marginally improve the outcome of such individuals, their median survival time is less than one year after diagnosis. A plateau has been reached in the efficacy of chemotherapy using nonspecific cytotoxic agents. Recent progress in understanding the biology of this tumor, characterization of NSCLC by molecular typing, particularly in adenocarcinomas of the lung, has led to the investigation of therapeutic agents that target dominant oncogenic mutations and show improved response rates in patients with NSCLCs [6-11]. In the recent years, several potential oncogenic drivers have been identified in patients with NSCLC, including epidermal growth factor receptor (EGFR), B-Raf (BRAF), Kirsten rat sarcoma 2 viral oncogene homolog (KRAS), mesenchymal epithelial transition factor (MET), human epidermal growth factor receptor 2 (HER2), and andanaplastic lymphoma kinase gene (ALK). Specifically, the discovery of the biologic and therapeutic importance of acquired genetic alterations in 2 genes that encode pharmacologically targetable tyrosine kinases involved in growth factor receptor signaling, epidermal growth factor receptor (EGFR) and anaplastic lymphoma kinase (ALK), has changed the way these cancers are diagnosed and treated. It is well known that mutations of the epidermal growth factor receptor (EGFR) gene are present in some patients with lung adenocarcinoma, with the implication that patients with this genotype were super responders to EGFR-tyrosine kinase inhibitors (TKIs), including gefitinib and erlotinib. In patients with a somatic EGFR mutation, frontline treatment with an EGFR inhibitor, gefitinib, results in improved response rate and superior progression-free survival. This benefit is limited to patients with EGFR mutation, as patients without the mutation have better overall response rates and progression-free survival with combination chemotherapy.

The anaplastic lymphoma kinase gene (ALK) is a member of the insulin receptor family and encodes a receptor tyrosine kinase that was originally identified in anaplastic large-cell lymphoma as a component of the fused protein NPM-ALK [12,13]. Subsequently, numerous other rearrangements that involve ALK have been identified, including TPM3-ALK, ATIC-ALK, TFG-ALK, CLTCALK and TPM4-ALK. In addition to anaplastic large-cell lymphoma, ALK fusion genes have also been described in half of the inflammatory myofibroblastic tumors and rare ALK-positive diffuse large B-cell lymphomas [14-17]. Recently, by screening a retroviral complementary DNA expression library generated from a NSCLC patient tumor sample, a rearrangements in the anaplastic lymphoma kinase gene (ALK) and echinoderm microtubule-associated protein-like 4 (EML4) have been identified as a result of a small inversion within the short arm of chromosome 2 between the N -terminal half of EML4 and the intracellular kinase domain of ALK [18]. EML4-ALK rearrangement has been demonstrated to be a potent oncogenic drive and a promising therapeutic target in NSCLC. It defines a distinct molecular subset of NSCLC, in particular adenocarcinoma that can benefit by the treatment of ALK-inhibitors [19-22]. EML4-ALK gene fusions occur in 2% to 7% of lung adenocarcinomas [23-26]. ALK rearrangement-positive patients treated with a novel ALK inhibitor, crizotinib, showed an overall response rate of 57%, with 72% having a PFS of 6 months or greater. The US Food and Drug Administration (FDA) has approved crizotinib for advanced-stage, ALK positive lung cancer as is also recommended by recent guidelines from professional organizations, including the American Society of Clinical Oncology (ASCO), European Society for Medical Oncology, and National Comprehensive Cancer Network (NCCN).

Despite the encouraging new target treatments have afforded benefits to patients with lung adenocarcinoma, but unfortunately the same is not true for lung SqCC [27,28]. Notably, the vast majority of ALK gene rearrangements were observed in lung adenocarcinoma specimens. The incidence of ALK gene rearrangements in lung SqCC has not been well documented except few cases were reported [29,30]. In the present study, we carried out an immunohistochemical staining in 207 cases of consecutive lung SqCC patients with a VENTANA anti-ALK (D5F3) specific rabbit monoclonal antibody. The positive samples were confirmed with ALK Fluorescence In Situ Hybridization (FISH).

## Materials and methods

### Clinical samples

Patients diagnosed with squamous cell lung carcinoma and the tumor specimens subjected to ALK protein expression analysis in Jinling Hospital, China, between 2010 and 2014 were included in this study (Additional file 1). A total of 207 consecutive cases of squamous cell lung carcinoma were retrieved from Department of Pathology. The clinicopathologic characteristics were obtained from medical records. The tissue samples included 135 cases of surgical resection samples and 72 cases of biopsy samples. The male of female ratio of the squamous lung carcinoma patients was 181/26. The median age of the patients was 63 years (range, 37–96 years). On the basis of World Health Organization Classification of Tumors (WHO), the Pathology and Genetics of Tumors of the Lung, Pleura, Thymus and Heart (2014), and International Association for the Study of Lung Cancer/American Thoracic Society/European Respiratory International Multidisciplinary Classification of Lung Adenocarcinoma [31], in 135 cases of surgical resection samples, 24 patients had stage I diseases, 31 patients had stage II diseases, 62 patients had stage III diseases, and 18 patients had stage IV diseases. The study protocol was approved by the Jinling Hospital Clinical Research Ethics Committee, China.

### Immunohistochemistry

Four μm-thick sections were cut from Formalin-fixed paraffin-embedded (FFPE) archive lung SqCC tissues blocks. Immunohistochemical staining was carried out according to the protocol provided by Ventana Medical Systems, Inc. and Roche Diagnostics International, Inc. Briefly, staining will require one serial tissue section for H&E, a second serial tissue section for VENTANA anti-ALK (D5F3), and a third serial tissue section for the Rabbit Monoclonal Negative Control Ig antibody. The assay uses the OptiView DAB IHC Detection Kit and OptiView Amplification Kit, and is run on the BenchMark ULTRA platforms. The VENTANA ALK 2 in 1 Control Slides are used as a system-level control. Staining was graded semiquantitatively, as follows: 0 for absent or barely perceptible expression in rare cells, 1 (low) for weak to moderate multifocal expression, and 2 (high) for strong staining in most cells. Positive cases stained with the VENTANA anti-ALK (D5F3) IHC assay typically display a strong, granular cytoplasmic signal. Known staining elements should be excluded, including: light cytoplasmic stippling in alveolar macrophages, cells of neural origin (nerve and ganglion cells), glandular epithelial staining, and cells within lymphocytic infiltrate. Some background staining also may be observed within normal mucosa in NSCLC (including mucin) and in necrotic tumor areas, which also should be excluded from the clinical evaluation.

### Fluorescence in Situ Hybridization (FISH)

Lung SqCC samples that positive for ALK IHC were subjected ALK FISH. Four μm-thick formalin-fixed, paraffin-embedded tissue sections were used for evaluation of ALK genetic status. All cases had interphase FISH performed for ALK rearrangement with the commercial ALK dual color, break-apart rearrangement probe (GP Medical Technologies, Beijing, China). Hybridization was carried out according to the protocol provided by the manufacturer. Briefly, formalin-fixed, paraffin-embedded samples were sectioned at a thickness of 4 μm. The sections were deparaffinized using xylene, dehydrated by gradient ethanol, and rehydrated with deionized water. The prepared slides were washed with 2x SSC, treated with 0.1 mol/L HCl, and digested with pepsin (P700; Sigma-Aldrich, St. Louis, MO). The slides and probe mixture were denatured separately. The denatured probe mixture was pipetted onto slides, and hybridization was performed in a wet box at 37°C overnight. After being washed, the air-dried slides were restained with 4’, 6-diamindino-2-phenylindole before being analyzed. One hundred individual interphase nuclei per specimen were enumerated based on localization in the corresponding H&E-stained sections. Samples were classified as positive for ALK rearrangement when 15% or more of nuclei showed split signals (i.e., red and green signals were separated by ≥2 signal diameters) or single red signals (3′ ALK) were observed. H&E and FISH slides for all cases were reviewed by two pathologists to confirm that scoring was carried out in the tumor cell population.

### EML4–ALK RT–PCR

Total RNA was extracted from the formalin-fixed, paraffin-embedded tissue with the RNeasy FFPE Kit (Qiagen, Hamburg, Germany). Reverse transcription and the detection of EML4–ALK were performed with the EML4–ALK Fusion Gene Detection Kit (AmoyDx, Xiamen, China). The primer sets used to amplify EML4–ALK variant 1, 3a/b, 2, 5′, and 5a/b are included in the kit. PCR was performed with the following parameters: denaturation at 95°C for 5 min; 15 cycles of 95°C for 25 s, 64°C for 20 s, and 72°C for 20 s; and 31 cycles of 93°C for 25 s, 60°C for 35 s, and 72°C for 20 s.

## Results

### ALK immunohistochemistry

**Figure 1:**
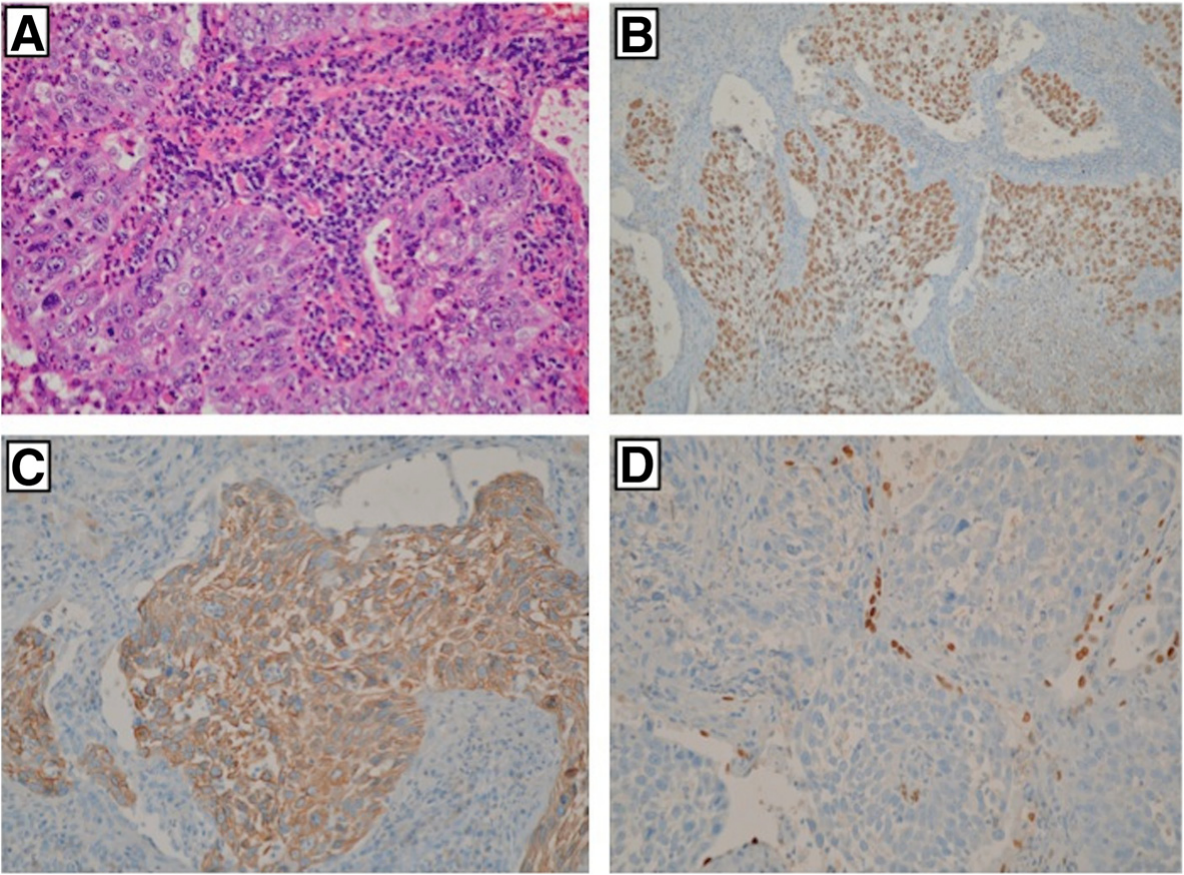
**Lung SqCC samples were diagnosed combining morphologic appearance and IHC staining including thyroid transcription factor-1 (TTF-1), transformation-related protein 63 (p63) and/or cytokeratin (CK5/6). A**: Hematoxlin and eosin staining. **B**: IHC staining for p63. **C**: IHC staining for CK5/6. **D**: IHC staining for TTF-1. Original magnification, ×400.

**Figure 2:**
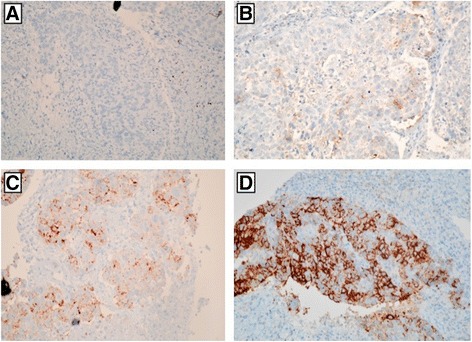
**Representative ALK IHC images. A**: Negative. **B**: Weak expression. **C**: Moderate expression. **D**: Strong staining. Original magnification, ×400.

All lung SqCC samples were diagnosed combining morphologic appearance and IHC staining including thyroid transcription factor-1 (TTF-1), transformation-related protein 63 (p63) and/or cytokeratin (CK5/6) (Figure 1). The intensity of staining ranges from weak, intermediate to strongly positive. Heterogeneous staining was noted from the same case. Of the 207 cases by immunohistochemistry, cytoplasmic expression of ALK was strongly (marker 2) observed in 3 cases with the VENTANA anti-ALK (D5F3), intermediately (marker 1) stained in 2 cases and weakly (marker 1) stained in 4 cases (Figure 2).

### ALK FISH

**Figure 3:**
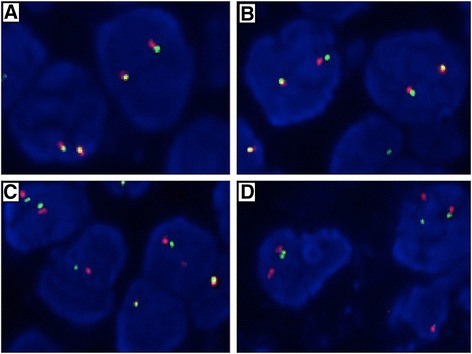
**Dual-color, break-apart fluorescent in situ hybridization was performed to confirm the ALK expression.** The centromeric (green) and telomeric (red) fland the ALK locus. Splitting of the red and green signals indicates ALK rearrangement. **A**: No EML4-ALK rearrangement. **B**, **C** and **D**: EML4-ALK rearrangements. A break-apart signal pattern, where one fusion signal and a single red and green signal pattern was observed in most nuclei **(B** and **C)**. A few nuclei showed a predominant signal pattern of deletion of the 5’region **(D)**.

All cases of SqCC samples with expression of ALK (marker 1 and 2) were confirmed by ALK FISH. A break-apart signal pattern, where one fusion signal and a single red and green signal pattern was observed in most nuclei. A few nuclei showed a predominant signal pattern of deletion of the 5’ region (nuclei with a single red signal in addition to a fused signal). Six case of lung SqCC with weak or intermediate staining of ALK showed FISH negative, while other 3 cases of lung SqCC with strong staining of ALK showed positive for ALK FISH (Figure 3).

### EML4–ALK rearrangement detected with RT–PCR

We investigated the presence of the EML4–ALK fusion variant in 30 samples of Sq CC using RT–PCR, which including 3 samples that were positive for IHC. The rearrangement-positive was confirmed in these three samples and rearrangement-negative was found in other samples.

## Discussion

Immunohistochemistry for ALK protein expression has been available for many years for use in the diagnosis of anaplastic large-cell lymphoma, but the traditional antibodies for use in lymphoma are insufficiently sensitive for detection at the level at which it is expressed in ALK-rearranged lung cancers. However, several recent studies have demonstrated that several relatively new ALK clones can accurately identify ALK-rearranged lung ACA as compared with FISH [2,8,29,32,33]. Studies published comparing the 5A4 antibody with ALK FISH demonstrated a sensitivity and specificity of 95% to 100%. Although strong staining seems to be 100% specific for the presence of rearrangement by FISH, weak-to-intermediate staining has been reported in FISH-negative tumors. In the present study, we detected 9 cases of positive expression of ALK, which including 3 strong staining, 2 intermediate staining and 4 weak staining. Three cases with strong staining were positive and other 6 cases were negative for ALK FISH. Although break-apart FISH is the standard method for diagnosis, it is expensive, not readily available and sometimes difficult to interpret. Immunohistochemistry for ALK protein overexpression is a promising screening modality, with newer antibodies showing excellent sensitivity and specificity for ALK-rearranged lung.

The echinoderm microtubule-associated protein-like 4 anaplastic lymphoma kinase (EML4-ALK) fusion gene has been identified as a potent oncogenic driver in non-small-cell lung cancer, in particular adenocarcinoma. It defines a distinct molecular subset of NSCLC that can benefit by the treatment of ALK-inhibitors. ALK rearrangements have been reported to occur at frequencies that range from 0.4 to 15% in NSCLC. Some series included patient populations likely to be enriched for ALK rearrangements, however, the average frequency in unselected NSCLC populations is around 3-4%. Though different groups investigated frequency of EML4-ALK fusion gene in NSCLC, among those NSCLC the sample number of lung SqCC was not large enough. People pay more attentions on lung adenocarcinoma than on lung SqCC. Eventually, some groups excluded SqCC from their study on EML4-ALK [2,10,19,34]. Can we eliminate squamous cell carcinoma of the lung from testing of EML4-ALK fusiong gene? Takigawa et al. recently raised the question [30]. They reported a 45 years old Japanese woman diagnosed as squamous cell carcinoma of lung harbored EML4-ALK gene rearrangement. Interestingly, Benepal et al. reported that an ALK rearrangement detected by FISH in a 69-year-old Caucasian gentleman diagnosed as lung squamous cell carcinoma [29]. Table 1 shows the prevalence of ALK positive SqCC all over the world [29,30,32,35].

**Table 1:** **ALK rearrangement study reports in squamous cell lung cancer patients**

**No.**	**Authors (reference No.)**	**Ethnic group**	**Patient number**	**Methods used in detection of ALK**			**ALK detection**	
				RT-PCR	IHC	FISH	Positive	Negative
1	Wu et al. [32]	Asian	75	Yes	Yes	ND	1(1.33%)	74(98.67%)
2	Alrifai et al. [29]	Caucasian	1	ND	ND	Yes	1	0
3	Ochi et al. [30]	Asian	1	ND	Yes	ND	1	0
4	Calio et al. [35]	Caucasian	40	ND	ND	Yes	1(2.5%)	39(97.5%)

RT-PCR: reverse transcriptase-polymerase chain reaction; FISH: fluorescence in situ hybridization; IHC: immunohistochemical stain; ND: not done.

Out results indicate that EML4-ALK fusion gene rearrangements occurred in 1.4% lung SqCC in Chinese population, which is not a rare molecular event and may have an impact on treatment choice or response to therapy. Although the frequency of these mutations is lower in lung SqCC than that in lung adenocarcinomas, their presence may provide additional treatment options in this group. The response of SqCC patients with ALK expression to target therapy of crizotinib should be explored. A more detailed clinicopathologic study, including examination of clinical outcomes in ALK positive versus carefully matched ALK negative patients, is in progress, results of which will be reported separately.

## Additional file

Additional file 1:
**The clinical pathological parameters of patients.**
